# Quantitative assessment of Pulmonary Alveolar Proteinosis (PAP) with ultra-dose CT and correlation with Pulmonary Function Tests (PFTs)

**DOI:** 10.1371/journal.pone.0172958

**Published:** 2017-03-16

**Authors:** Xin Sui, Qianni Du, Kai-feng Xu, Xinlun Tian, Lan Song, Xiao Wang, Xiaoli Xu, Zixing Wang, Yuyan Wang, Jun Gu, Wei Song, Zhengyu Jin

**Affiliations:** 1 Department of Radiology, Peking Union Medical College Hospital, Chinese Academy of Medical Sciences, Beijing, China; 2 Department of Respiratory Medicine, Peking Union Medical College Hospital, Chinese Academy of Medical Sciences, Beijing, China; 3 Department of Epidemiology and Biostatistics, Institute of Basic Medical Science, Chinese Academy of Medical Sciences & School of Basic Medicine, Peking Union Medical College, Beijing, China; 4 Siemens Healthineers, Beijing, China; Universitatsklinikum Freiburg, GERMANY

## Abstract

**Background:**

The purpose of this study was to investigate whether ultra-low-dose chest computed tomography (CT) can be used for visual assessment of CT features in patients with pulmonary alveolar proteinosis (PAP) and to evaluate the relationship between the quantitative analysis of the ultra-low-dose CT scans and the pulmonary function tests (PFTs).

**Methods:**

Thirty-eight patients (mean [SD] age, 44.47 [12.28] years; 29 males, 9 females) with PAP were enrolled and subjected to two scans each with low-dose CT (reference parameters: 120 kV and 50 mAs) and ultra-low-dose CT (reference parameters, 80 kV, 25 mAs). Images were reconstructed via filtered back projection (FBP) for low-dose CT and iterative reconstruction (IR) for ultra-low-dose CT. All patients underwent PFT. The Visual analysis for ground glass opacity (GGO) is performed. The quantitative CT and PFT results were analyzed by canonical correlations.

**Results:**

The mean body mass index (BMI) was 25.37±3.26 kg/m^2^. The effective radiation doses were 2.30±0.46 and 0.24±0.05 mSv for low-dose and ultra-low-dose CT, respectively. The size-specific dose estimates were 5.81±0.81 and 0.62±0.09 mSv for low-dose and ultra-low-dose CT. GGOs and interlobular septal thickening were observed bilaterally in all patients. The average visual GGO score was lower in the upper field (2.67±1.24) but higher in the middle and lower fields (3.08±1.32 and 3.08±0.97, respectively). The average score for the whole lung was 2.94±1.19. There is a significant correlation between PFTs and quantitative of ultra-low-dose CT (canonical loading = 0.78).

**Conclusions:**

Ultra-low-dose CT has the potential to quantify the lung parenchyma changes of PAP. This technique could provide a sensitive and objective assessment of PAP and has good relation with PFTs. In addition, the radiation dose of ultra-low-dose CT was very low.

## Introduction

Pulmonary alveolar proteinosis (PAP) is caused by an intra-alveolar accumulation of lipoproteinaceous material [1]. Chest CT imaging characteristics of PAP include interlobular septal thickening and ground glass opacity (GGO), described as a “crazy-paving” pattern [2]. It is difficult to precisely estimate air space opacities to monitor the development of PAP on conventional CT. Quantitative CT detects the changes of lung density caused by disease progression, which are related to the attenuation of x-rays. Therefore, quantitative CT with a standard radiation dose is an objective tool that can be used to assess accurate morphological changes, the therapeutic response [3, 4] and long-term follow-up [5, 6].

However, previous quantitative analyses have been conducted on standard chest CT scans with an effective dose of 6–8 mSv [7] which is too high especially for the young individuals. Moreover repeated CT scans for follow-up with such a high dose will lead to radiation accumulation. For the potential increase in the risk of radiation-induced carcinogensis, the radiation dose delivered by CT scanning has drawn increasing attention [8]. Hence reducing the radiation dose will be of great benefits to patients. However, the clinic application of low dose CT (LDCT) focuses mainly on lung cancer screening [9, 10]. There is a question, in addition to nodule detection, how much dose reduction is reasonable for assessment of lung diseases. Low-dose CT is quantitatively accurate in detecting disease progression for patients with emphysema by analysis of low-attenuation [6, 11]. Those studies focused on the decreased lung attenuation disease.

Compared with decreased lung attenuation, increased lung attenuation is in a way by opposite phenomena and no published study has evaluated the efficacy of low-dose CT as a quantitative measure for patients with lesions of high attenuation. The specific CT patterns of PAP are GGO, interlobular septal thickening and the “crazy-paving” pattern for partial filling of the alveolar spaces [2]. The current prospective study investigated whether ultra-low-dose CT scans can be used for visual assessment of CT features in patients with PAP and to evaluate the relationship between the quantitative analysis of the ultra-low-dose CT scans and the pulmonary function tests (PFTs). Devising ways to reduce radiation exposure during chest CT scans will greatly benefit for PAP patients.

## Materials and methods

### Participants

This single-center, prospective, observational study is approved by the Institutional Review Board of Peking Union Medical College Hospital (Approval No. S-598). Patients with PAP (diagnosed by transbronchial lung biopsies, chest imaging, and clinical manifestations) were enrolled in this study between November 2014 and March 2016. Before CT scans, no whole lung lavages were performed. Written informed consent to participate in this study was obtained from each participant or their family members when the patient was incapable of consent.

The procedure, which involved LDCT acquisition followed by ultra-low-dose CT, was explained to the participants. They were also informed that the radiation dose from the ultra-low-dose CT was comparable with the cumulative dose of a chest radiograph. All patients received PFTs within 0–7 days after their chest CT scan.

### CT protocol

All CT scans were performed using a 128-section dual-energy CT system (SOMATOM Definition Flash, Siemens Healthcare, Forchheim, Germany) equipped with high-resolution circuit detectors known as Stellar. The examinations were performed with a gantry rotation time of 0.28 s, pitch = 1.5, and 2×128×0.6 mm collimation width with a z-flying focal spot. Attenuation-based tube current and tube voltage modulations (CARE Dose 4D and CARE kV) used settings that were optimized for non-contrast examinations to ensure a similar noise index for each participant. According to the image quality reference mAs and kV, the scanner adapted the tube current for each scan position based on the size of the participant to obtain the same target image quality (as defined by the quality reference kV and mAs) throughout the scan. The tube voltage, which was based on the reference kV, was adapted to patient body size throughout the scan. The reference kV and mA values were 120 kV/50 mAs and 80 kV/25 mAs for LDCT and ultra-low-dose CT, respectively. The scan ranged from the costophrenic angle to the pulmonary apex. Participants were instructed to hold their breath in deep inspiration during the CT examination.

### Image reconstruction

Both LDCT and ultra-low-dose CT data were reconstructed using standard filtered back projection (FBP) with a soft kernel (B30f), with reconstructed a 1.0-mm slice thickness and 1.0-mm increment. In addition, ultra-low-dose CT data were reconstructed with iterative reconstruction (IR), namely sinogram-affirmed IR (SAFIRE) at a strength of 3 with a soft kernel (I30f). Previous study showed that the mean lung attenuation was not change (less than 2 HU) with reconstruction algorithm and slice thickness [12]. The ultra-low-dose CT images of two kinds of reconstruction algorithms (FBP and IR) were used for visual assessment.

### Radiation dose assessment

The dosage parameters generated from both the LDCT and ultra-low-dose CT protocols were recorded as the CT dose index-volume (CTDIvol) and the dose-length product (DLP). The effective dose (ED) was calculated by multiplying the DLP by a chest conversion coefficient (k: 0.014 mSv/mGy*cm) [13]. For each patient, the data of anteroposterior (AP) thickness at the midline and lateral (LAT) width was measured at the liver level from axial CT image. In the light of the chest $effective\;diameter=\sqrt{\left(AP\cdot LAT\right)}$ [14], size-specific dose estimates (SSDEs) were evaluated by the size-specific conversion factor (*fsize*) from AAPM Report 204 [15]. The specific formula was defined: *SSDE* = (*fsize*)·CTDIvol.

### Image noise

Image noise was assessed by measuring the standard deviation of regions of interest (ROI) placed by a radiologist with 6 years of experience in CT. ROIs were drawn at the air of tracheal lumen above the aortic arch. The ROI was defined as encompassing an area of 1 cm^2^. CT attenuation was measured in images of LDCT with FBP and ultra-low dose CT with FBP and IR. Mean image noise was defined as the mean of the standard deviation of the attenuation value in consecutive ROI measurements.

### Quantitative CT analysis

#### Visual analysis

Images were viewed with both lung (window center -600 HU, width = 1,200 HU) and mediastinal (window center -40 HU, width = 400 HU) window settings. Two thoracic radiologists with 10 and 6 years of experience in thoracic imaging conducted the analysis. These radiologists independently evaluated the extent of pulmonary abnormalities. The CT images were judged for GGO, consolidation, interlobular septal thickening and honeycombing.

The area was evaluated for GGOs in six zones. The upper zone was defined as the part of the lung above the aortic arch. The middle zone was defined as the area between the aortic arch and the pulmonary veins, and the lower zone was defined as the area below the pulmonary veins. [16] The visual scores were defined as follows: grade 0, no opacity; grade 1, 5% opacity; grade 2, 5% to 24% opacity; grade 3, 25% to 49% opacity; grade 4, 50% to 74% opacity; and grade 5, 75% opacity (S1–S5 Figs).

#### Automatic analysis

The acquired CT data were post-processed with Pulmo 3D (syngo.via, version VA 30, Siemens Healthcare, Germany) for the automatic segmentation of the pulmonary parenchyma by excluding the intrapulmonary vessels. The total lung volume and mean lung attenuation were automatically calculated. The lung density (grams per milliliter) was estimated by adding 1,000 to the HU of each voxel and then dividing by 1,000 [17]. Lung weight was calculated by multiplying the lung density of each voxel by the voxel volume. The first radiologist independently measured the quantitative CT data. To minimize observer influence, no manual interaction was allowed during the correction of segmentation errors. Inter-scan variability was derived from the measurements.

### Pulmonary function tests

PFT measurements, including forced vital capacity (FVC), forced expiratory volume in 1 second (FEV_1_), FEV_1_/FVC, diffusing capacity for carbon monoxide (D_LCO_), and D_LCO_ corrected for alveolar volume (D_LCO_/VA), were calculated. These values were determined based on the standards of the American Thoracic Society, and the results are shown as the percentages of the predicted values using accepted standard formulas.

### Statistical analyses

Continuous variables were reported as the means±standard deviations (SDs). Numerical data were evaluated for normal distributions using the Doornik-Hansen test. Parametric data were compared using the t-test for two-group comparisons. Inter-rater agreement was assessed using the Kappa statistic. Inter-rater agreement was classified as follows: poor, Kappa = 0 to 0.20; fair, Kappa = 0.21 to 0.40; moderate, Kappa = 0.41 to 0.60; good, Kappa = 0.61 to 0.80; and excellent, Kappa = 0.81 to 1.00. A one-way repeated-measures ANOVA was used to compare the quantitative measurements among the three reconstructed image groups.

This study investigated the potential association between pulmonary function and CT parameters. Canonical correlation was used to explore the relationship between two sets of variables [18]. The input (FEV_1_%, FVC%, FEV_1_/FVC, D_LCO_%, D_LCO_/VA) and output variables (total lung volume, lung weight, mean lung density) were compared to determine the coefficients (a and b) that maximized the correlation between the canonical variates (Vi and Wi). The canonical solutions consisted of a linear combination of canonical variables formed by

$$V_{i}=a_{1}\left(input_{1}\right)+a_{2}\left(input_{2}\right)+\cdots +a_{n}\left(input_{n}\right)$$ (1)

$$W_{i}=b_{1}\left(output_{1}\right)+b_{2}\left(output_{2}\right)+\cdots +b_{n}\left(output_{n}\right)$$ (2)

The first canonical correlation (U1, U2) represents the highest possible correlation between any linear combination of input variables and any linear combination of output variables. A P-value of <0.05 was considered as significantly different. SAS (version 9.4, for Windows) was used to analyze the data.

## Results

A total of 40 patients were enrolled during the inclusion period. Two patients were excluded because the automated lung segmentation conducted using Pulmo 3D failed. The visual scores of the two patients are 5. The lung density was too high to segment the parenchyma from the intrapulmonary vessels. Thus, a final sample of 38 patients was included. The ages, genders, heights, weights, body mass indices (BMIs), PFTs and radiation dosages for the patients are shown in Table 1. There are no underweight patients (BMI <18.5 kg/m^2^). There are 21 normal patients (BMI between 18.5 and 24.9 kg/m^2^), 15 over-weight patients (BMI between 25 and 29.9 kg/m^2^) and 2 obese patients (BMI >30 kg/m^2^). The DLPs were 163.94±33.06 mGy*m on LDCT and 17.47±3.47 mGy*m on ultra-low-dose CT (P<0.001). The EDs were 2.30±0.46 mSv on LDCT and 0.24±0.05 mSv on ultra-low-dose CT (P<0.001). The SSDEs were 5.81±0.81 mGy on LDCT and 0.62±0.09 mGy on ultra-low-dose CT (P<0.001). All patients showed abnormalities in the percentages of predicted FEV_1_ and D_LCO_.

**Table 1:** Patient clinical characteristics and radiation dose.

Characteristics of patients, PFT and radiation dose	
Age(years), mean±S.D., (range)	44.47±12.28 (20–61)
Men/Women	29/9
Height (m), mean±S.D., (range)	1.69±0.07 (1.5–1.85)
Body weight (kg), mean±S.D., (range)	73.05±11.68 (48–95)
BMI (kg/m2), mean±S.D.	25.37±3.26
PFTs	
FEV_1_%	78.57±15.60
FVC	81.46±15.00
FEV_1_/FVC%	95.59±8.51
D_LCO_%	64.82±14.44
D_LCO_/VA%	89.89±17.93
DLP (mGy×cm), mean±S.D (LDCT)	163.94±33.06
DLP (mGy×cm), mean±S.D (Ultra-low-dose CT)	17.47±3.47
ED (mSv), mean±S.D. (LDCT)	2.30±0.46
ED (mSv), mean±S.D. (Ultra-low-dose CT)	0.24±0.05
SSDE(mGy), mean±S.D (LDCT)	5.81±0.81
SSDE(mGy), mean±S.D (Ultra-low-dose CT)	0.62±0.09

BMI: Body mass index

PFT: Pulmonary function test

FEV_1_: forced expiratory volume in 1 second

FVC: forced vital capacity

D_LCO_: diffusing capacity for carbon monoxide

D_LCO_/VA: diffusing capacity for carbon monoxide corrected for alveolar volume

DLP: Dose length product

SSDE: Size-specific dose estimate

ED: Effective dose

Compared to LDCT with FBP (17.92 ± 5.73) HU, the image noise was higher for ultra-low-dose CT with FBP and IR (P < 0.001). The image noise of ultra-low-dose CT reconstructed with IR was (23.24 ± 4.23) HU, lower than (35.05 ± 6.39) HU on ultra-low-dose with FBP (P < 0.001). The image noise of ultra-low-dose CT with IR was much lower than that with FBP.

The PAP lesions were characterized based on GGOs, interlobular septal thickening as observed on both LDCT and ultra-low-dose CT scans. No honeycombing was observed for any case. GGOs and interlobular septal thickening were observed bilaterally in all patients. Emphysema was observed in 1 patient. To evaluate the distribution of GGOs, the proportion of the involved GGO area was determined using a visual scoring system for the upper, middle, and lower lung fields. The average score was lower in the upper field (2.67±1.24) but higher in the middle and lower fields (3.08±1.32 and 3.08±0.97, respectively). The average visual GGO score for the whole lung was 2.94±1.19. The inter-observer agreement between the two radiologists for the low-dose CT was satisfactory (K = 0.75, 0.80). Better inter-observer agreement was achieved when using ultra-low-dose CT with IR (satisfactory: K = 0.64–0.69) compared with FBP (marginal: K = 0.50–0.61; Table 2).

**Table 2:** Diagnostic confidence in CT findings from patients with PAP in LDCT and ultra-low-dose CT.

Findings	Total PAP	K statistic (LDCT)	P value	K statistic (ultra-low-dose CT)			
				FBP	P value	IR	P value
GGO	38	0.80	<0.001	0.61	<0.001	0.69	<0.001
interlobular septal thickening	38	0.75	<0.001	0.50	<0.001	0.64	<0.001
honeycombing	0	1	1	1	1	1	1
Emphysema	1	1	1	1	1	1	1

GGO: ground glass opacity

FBP: filtered back projection

IR: iterative reconstruction

The quantitative measurements obtained from ultra-low-dose CT images reconstructed with FBP and IR were strongly correlated with the measurements derived from the LDCT images in terms of total lung volume, lung weight and mean lung density (Table 3).

**Table 3:** Quantitative assessments with low-dose and ultra-low-dose CT.

Value	LDCT	Ultra-low-dose CT		P (LDCT vs Ultra-low-dose CT)	
		FBP	IR	FBP	IR
Total lung volume (ml)	4598.84±960.21	4582.56±995.96	4602.34±978.51	0.95	0.99
Mean lung density (g/ml)	0.28±0.06	0.28±0.06	0.28±0.06	0.83	0.83
Lung weight (g)	1266.25±325.01	1275.85±333.93	1281.73±332.22	0.90	0.85

FBP: filtered back projection

IR: iterative reconstruction

All the PFTs and CT data demonstrated multivariate normal distributions (P = 0.37). The relationships between pulmonary function (inputs) and LDCT or ultra-low-dose CT with FBP/IR (outputs) across the 38 patients were analyzed via a canonical correlation analysis, which returned the first canonical correlation coefficients of 0.83 (P = 0.0007), 0.83 (P = 0.0009), and 0.82 (P = 0.0017). The correlations among quantitative CT, applied LDCT and ultra-low-dose CT with FBP, SAFIRE and PFTs are displayed in Table 4. The mean lung density was significantly correlated with D_LCO_ (canonical loading = 0.77–0.78; Table 4).

**Table 4:** Canonical loadings for input and output variates for patients with PAP.

Input set	V1			Output set	W1		
	LDCT	Ultra-low-dose CT			LDCT	Ultra-low-dose CT	
	FBP	FBP	IR		FBP	FBP	IR
FEV_1_%	0.47	0.48	0.46	Total lung volume	0.36	0.36	0.36
FVC%	0.60	0.58	0.57	Lung weight	-0.34	-0.35	-0.37
FEV_1_/FVC	-0.10	-0.06	-0.07	Mean lung density	-0.77	-0.78	-0.78
D_LCO_%	0.76	0.76	0.75				
D_LCO/_VA	0.33	0.35	-0.34				

FBP: filtered back projection

IR: iterative reconstruction

FVC: forced vital capacity

FEV_1_: forced expiratory volume in 1 second

D_LCO_: diffusing capacity for carbon monoxide

D_LCO_/VA: diffusing capacity for carbon monoxide corrected for alveolar volume

## Discussion

In this study, the mean effective radiation dose on ultra-low-dose CT for PAP was only 0.25mSv, which was close to a chest radiography (0.05–0.2 mSv) [19, 20]. In addition, the quantitative CT assessments were strongly correlated with PFTs. To the best of our knowledge, this study was the first to evaluate the potential of quantitative ultra-low-dose CT in patients with PAP.

Because of the high inherent contrast in the chest and the lower radiation absorption in pulmonary tissues, it is feasible to substantially reduce dose on chest CT [21, 22]. And LDCT might be useful to examine abnormalities that have high contrast within normal lung areas [23, 24]. Images on ultra-low-dose CT have yielded diagnostic quality for 93–97% of increased-attenuation lesions [23]. Previous studies manifested that the parameters obtained from quantitative standard dose CT were good correlated with PFTs for patients with PAP [3, 4, 25] and provided information about changes in lung volume and density. However, those studies used standard CT (120–140 kV, 155–200 mA), which resulted in much more radiation exposure than those obtained in our study (120 kV/50 mAs for LDCT and 80 kV/25 mAs for ultra-low-dose CT). Compared with the dose of standard CT which is 6–8 mSv [7], it is reduced by 96~97% on ultra-low-dose CT with the high resolution detector. Consequently, ultra-low-dose CT had great benefit and potential for the patients with PAP, especially for young patients and follow-ups for treatment.

FBP reconstruction is the standard reconstruction algorithm, but propagating image noise remains its major drawback. Therefore various types of IR techniques are generated to reduce radiation dose by the repeated subtraction of quantum noise and artifact elimination [26, 27]. IR improves the detection of conspicuous lesions [26]. SAFIRE is one of the most recently introduced IR methods, and it uses a noise modeling technique based on the raw data to tradeoff between reducing noise and maintaining image sharpness [26]. Our results showed that image noise reconstructed with IR was significantly lower than that reconstructed with FBP on ultra-low-dose CT, approaching to that reconstructed with FBP on LDCT. By reducing the image noise, IR improved the diagnostic confidence of ultra-low-dose CT. The inter-observer agreement about GGO and interlobular septal thickening was better with IR than FBP on ultra-low-dose CT in our study.

Cazy-paving is characteristic CT finding of PAP, but it could be observed in other diseases, such as diffuse alveolar damage (adult respiratory distress syndrome), lymphangitic carcinomatosis, pulmonary edema (causing by left heart failure). A great proportion of PAP are smokers. Some of patients with PAP had mild symptom but with diffuse lesions in CT images. Crucial diagnosis of PAP is representive by transbronchial or surgical lung biopsy, but sputum or Bronchoalveolar lavage can also be applied for diagnosis [28]. Since most of patients with PAP required intervention and rare patients progress to pulmonary fibrosis, it is indispensable to clinical and CT imaging follow-ups. For the radiation dose accumulation by follow-ups, it is great benefit to those patients by reducing radiation dose.

Chest CT scans play a major role in diagnosis of lung diseases and follow-ups. Quantitative CT might be an effective way to detect the extent of disease progression following treatment [3, 25]. In this study, the specific CT patterns were observed clearly on ultra-low-dose CT. Meanwhile the visual score for the average distribution in GGO was approximately 3 (25–49% opacity). The typical abnormality of PFTs in PAP is a decreased D_LCO_ [3, 25]. In this study, the mean lung density was clearly correlated with the D_LCO_ in LDCT and ultra-low-dose CT. It was the first to demonstrate the quantitative ultra-low-dose CT strongly associated with PFTs.

It is necessary to follow up patients with PAP to monitor the recurrence and treatment response such as whole lung lavages, aerosolized granulocyte-macrophage colony-stimulating factor. Repeated CT scans, which necessitate additional exposure to radiation, are undesirable. So patients with PAP will get benefit from ultra-low-dose CT for follow-ups. The quantitative analysis on ultra-low-dose CT might also be useful for other pulmonary diseases, including hypersensitivity pneumonitis, the early stages of usual interstitial pneumonia, collagen vascular disease, and drug-related lung disease. Although those extending clinic applications needs further studies, this approach might be suitable for patients who require a substantial dose reduction, such as young patients and those who need long-term follow-ups to evaluate their response to treatment response.

Certain limitations of this study merit consideration. First, we didn’t set a control group because PAP is a rare lung disease and the number of patients was small [1, 29]. And the radiation dose of ultra-low-dose CT is closing to the dose of chest radiograph. Second, regarding radiation dosage, we used LDCT as a reference instead of standard CT. Several studies [30–32] concluded that image quality did not differ between LDCT (40–50 mA) and standard CT.

Despite these limitations, this study found that ultra-low-dose CT with a high-resolution circuit detector had the potential to quantify the lung parenchyma changes of PAP. Ultra-low-dose CT might provide a sensitive and objective assessment of PAP. The measurements were good correlated with PFTs, and the radiation dose was substantially reduced. This method might be a particularly relevant option for follow-ups, especially for young patients who are more fragile and vulnerable to accumulated dose of radiation.

## Supporting information

S1 Fig The visual score was 1 (smaller than 5% opacity). A 33-year-old man with PAP. LDCT images with FBP (A), ultra-low-dose CT with FBP (B) and ultra-low-dose CT with IR (C).
(TIF)

S2 Fig The visual score was 2 (5% to 24% opacity). A 50-year-old man with PAP. LDCT images with FBP (D), ultra-low-dose CT with FBP (E) and ultra-low-dose CT with IR (F).
(TIF)

S3 Fig The visual score was 3 (25% to 49% opacity). A 28-year-old man with PAP. LDCT images with FBP (G), ultra-low-dose CT with FBP (H) and ultra-low-dose CT with IR (I).
(TIF)

S4 Fig The visual score was 4 (50% to 75% opacity). A 40-year-old man with PAP. LDCT images with FBP (J), ultra-low-dose CT with FBP (K) and ultra-low-dose CT with IR (L).
(TIF)

S5 Fig The visual score was 5 (larger than 75% opacity). A 36-year-old man with PAP. LDCT images with FBP (M), ultra-low-dose CT with FBP (N) and ultra-low-dose CT with IR (O).
(TIF)

S1 Table Patient clinical characteristics and radiation dose. (DOCX)

S2 Table Diagnostic confidence in CT findings from patients with PAP in LDCT and ultra-low-dose CT. (DOCX)

S3 Table Quantitative assessments with low-dose and ultra-low-dose CT. (DOCX)

S4 Table Canonical loadings for input and output variates for patients with PAP. (DOCX)
