# Supplementary figures and images for: Quantitative assessment of Pulmonary Alveolar Proteinosis (PAP) with ultra-dose CT and correlation with Pulmonary Function Tests (PFTs)
Source: PLoS One. 2017 Mar 16;12(3):e0172958. doi: 10.1371/journal.pone.0172958 (PMC5354367; doi:10.1371/journal.pone.0172958)

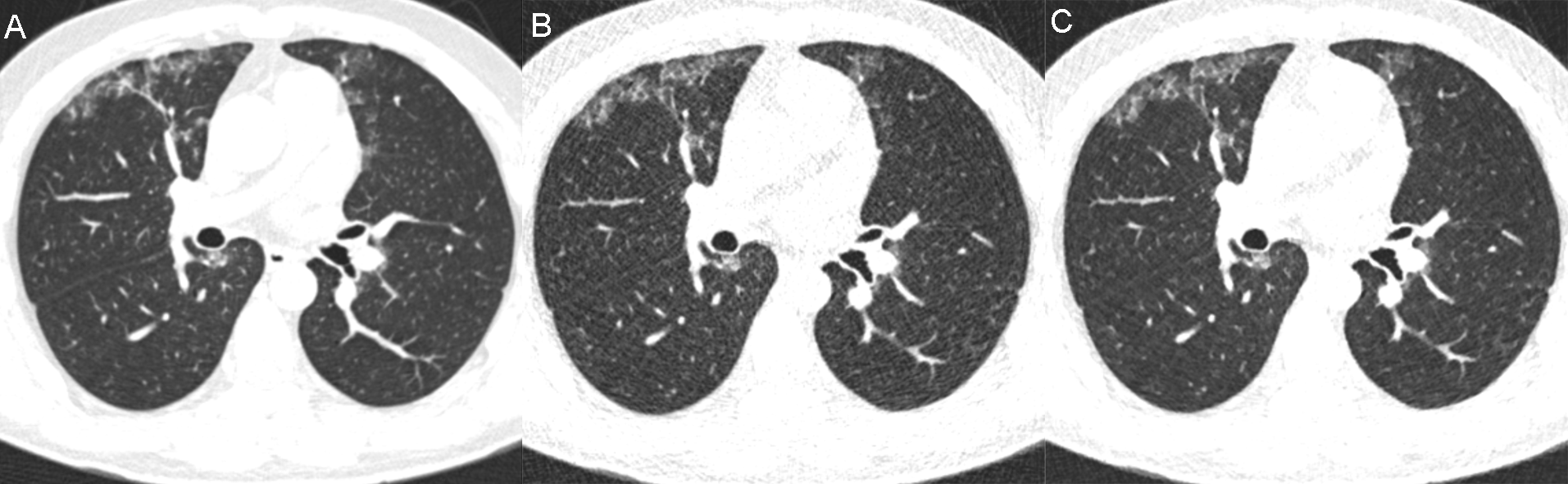

Supplement: S1 Fig — A 33-year-old man with PAP. LDCT images with FBP (A), ultra-low-dose CT with FBP (B) and ultra-low-dose CT with IR (C). (TIF) [file pone.0172958.s001.tif]

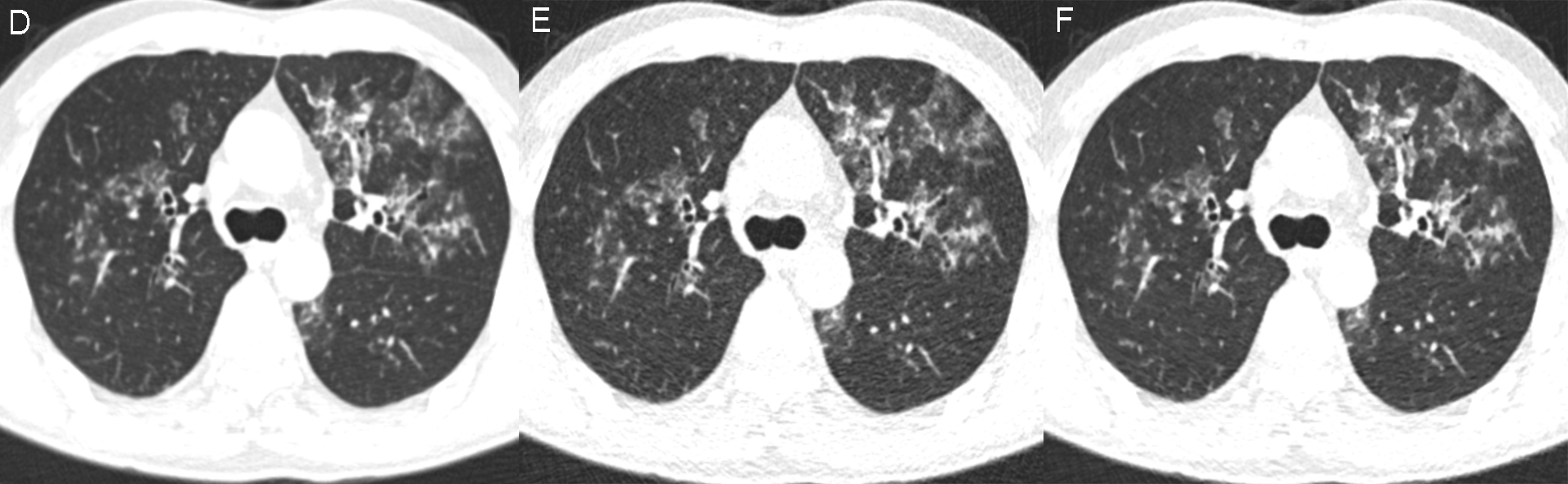

Supplement: S2 Fig — A 50-year-old man with PAP. LDCT images with FBP (D), ultra-low-dose CT with FBP (E) and ultra-low-dose CT with IR (F). (TIF) [file pone.0172958.s002.tif]

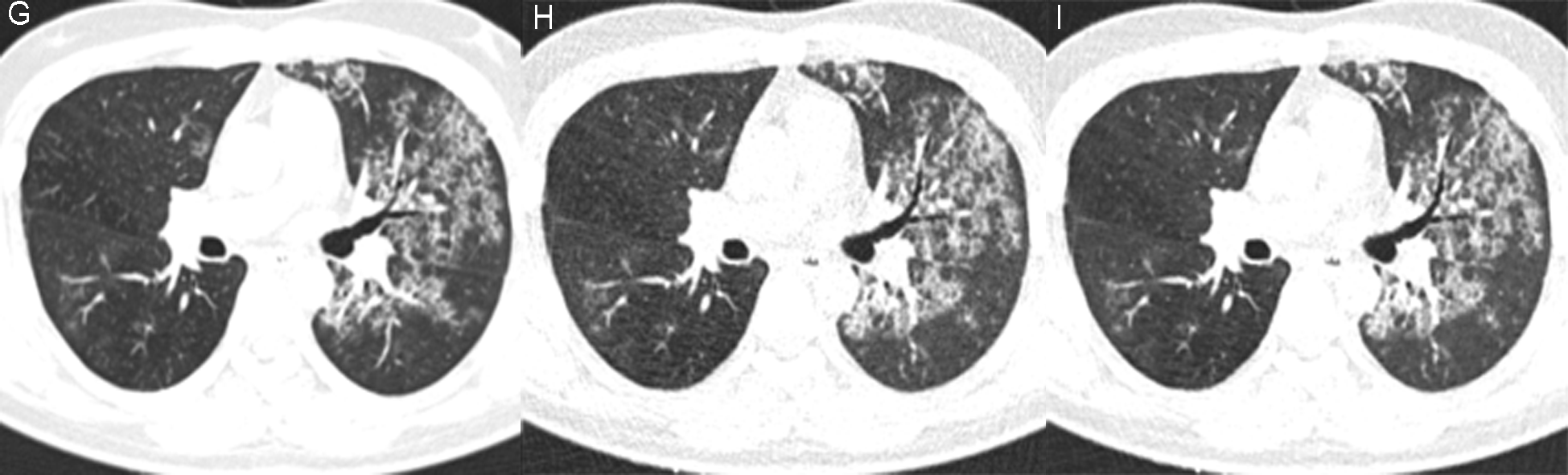

Supplement: S3 Fig — A 28-year-old man with PAP. LDCT images with FBP (G), ultra-low-dose CT with FBP (H) and ultra-low-dose CT with IR (I). (TIF) [file pone.0172958.s003.tif]

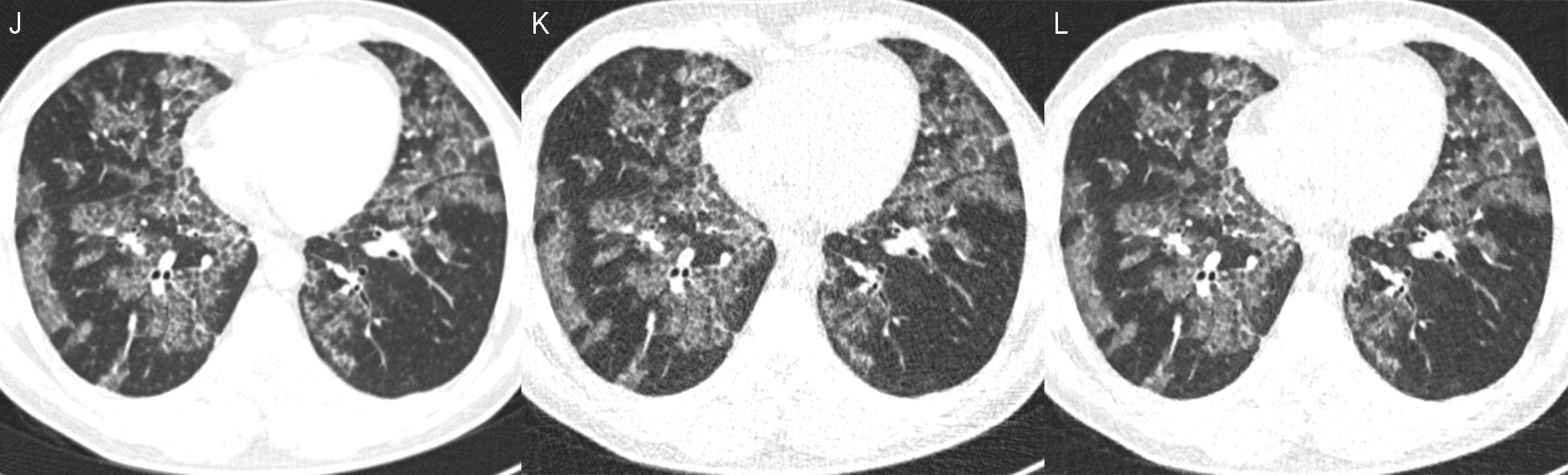

Supplement: S4 Fig — A 40-year-old man with PAP. LDCT images with FBP (J), ultra-low-dose CT with FBP (K) and ultra-low-dose CT with IR (L). (TIF) [file pone.0172958.s004.tif]

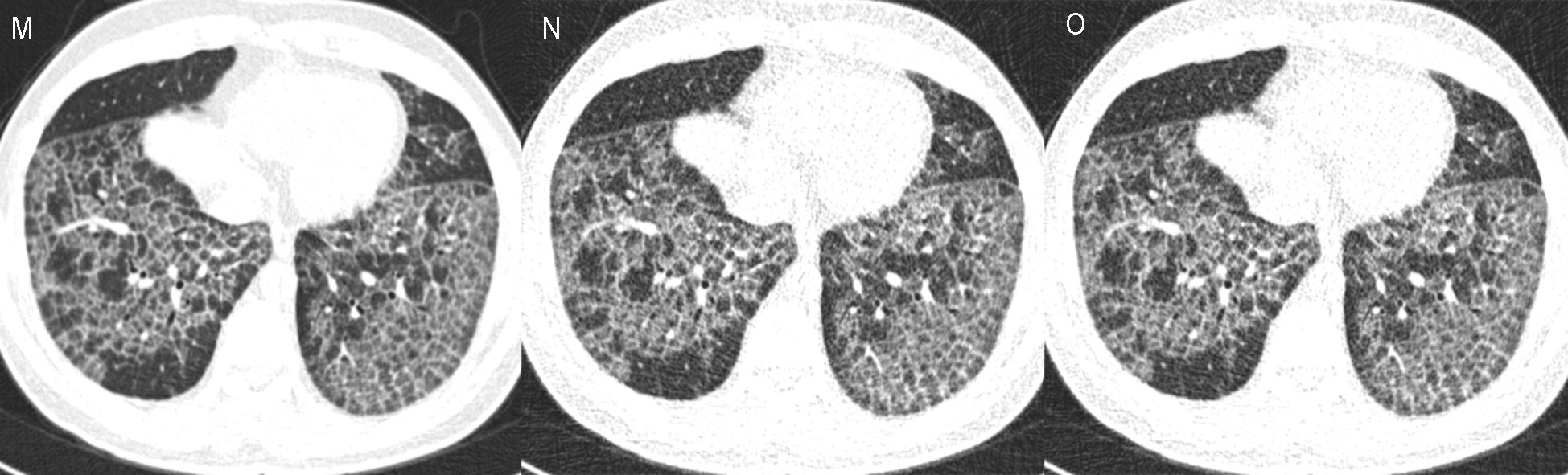

Supplement: S5 Fig — A 36-year-old man with PAP. LDCT images with FBP (M), ultra-low-dose CT with FBP (N) and ultra-low-dose CT with IR (O). (TIF) [file pone.0172958.s005.tif]
