# Supplementary material for: Quantitative assessment of Pulmonary Alveolar Proteinosis (PAP) with ultra-dose CT and correlation with Pulmonary Function Tests (PFTs)
Source: PLoS One. 2017 Mar 16;12(3):e0172958. doi: 10.1371/journal.pone.0172958 (PMC5354367; doi:10.1371/journal.pone.0172958)
Supplement: S1 Table — (DOCX) [file pone.0172958.s006.docx]

**Table 1.** Patient clinical characteristics and radiation dose

| Characteristics of patients, PFT and radiation dose | |
| --- | --- |
| Age(years), mean±S.D., (range) | 44.47±12.28 (20-61) |
| Men/Women | 29/9 |
| Height (m), mean±S.D., (range) | 1.69±0.07 (1.5-1.85) |
| Body weight (kg), mean±S.D., (range) | 73.05±11.68 (48-95) |
| BMI (kg/m2), mean±S.D. | 25.37±3.26 |
| PFT |  |
| FEV_1_% | 78.57±15.60 |
| FVC | 81.46±15.00 |
| FEV_1_/FVC% | 95.59±8.51 |
| D_LCO_% | 64.82±14.44 |
| D_LCO_/VA% | 89.89±17.93 |
| DLP (mGy×cm), mean±S.D (LDCT) | 163.94±33.06 |
| DLP (mGy×cm), mean±S.D (Ultra-low-dose CT) | 17.47±3.47 |
| ED (mSv), mean±S.D. (LDCT) | 2.30±0.46 |
| ED (mSv), mean±S.D. (Ultra-low-dose CT) | 0.24±0.05 |
| SSDE(mGy) , mean±S.D (LDCT) | 5.81±0.81 |
| SSDE(mGy) , mean±S.D (Ultra-low-dose CT) | 0.62±0.09 |

BMI: Body mass index

PFT: Pulmonary function test

FEV_1_: forced expiratory volume in 1 second

FVC: forced vital capacity

D_LCO_: diffusing capacity for carbon monoxide

D_LCO_/VA: diffusing capacity for carbon monoxide corrected for alveolar volume

DLP: Dose length product

SSDE: Size-specific dose estimate

ED: Effective dose
