# Supplementary material for: Quantitative assessment of Pulmonary Alveolar Proteinosis (PAP) with ultra-dose CT and correlation with Pulmonary Function Tests (PFTs)
Source: PLoS One. 2017 Mar 16;12(3):e0172958. doi: 10.1371/journal.pone.0172958 (PMC5354367; doi:10.1371/journal.pone.0172958)
Supplement: S2 Table — (DOCX) [file pone.0172958.s007.docx]

**Table 2.** Diagnostic confidence in CT findings from patients with PAP in LDCT and ultra-low-dose CT

| Findings | Total PAP | K statistic  (LDCT) | P value | K statistic (ultra-low-dose CT) | | | |
| --- | --- | --- | --- | --- | --- | --- | --- |
|  |  |  |  | FBP | P value | IR | P value |
| GGO | 38 | 0.80 | <0.001 | 0.61 | <0.001 | 0.69 | <0.001 |
| interlobular septal thickening | 38 | 0.75 | <0.001 | 0.50 | <0.001 | 0.64 | <0.001 |
| honeycombing | 0 | 1 | 1 | 1 | 1 | 1 | 1 |
| Emphysema | 1 | 1 | 1 | 1 | 1 | 1 | 1 |

GGO: ground glass opacity

FBP: filtered back projection

IR: iterative reconstruction
