# Supplementary material for: Quantitative assessment of Pulmonary Alveolar Proteinosis (PAP) with ultra-dose CT and correlation with Pulmonary Function Tests (PFTs)
Source: PLoS One. 2017 Mar 16;12(3):e0172958. doi: 10.1371/journal.pone.0172958 (PMC5354367; doi:10.1371/journal.pone.0172958)
Supplement: S3 Table — (DOCX) [file pone.0172958.s008.docx]

**Table 3.** Quantitative assessments with low-dose and ultra-low-dose CT

| Value | LDCT | Ultra-low-dose CT |  | P  (LDCT vs Ultra-low-dose CT) | |
| --- | --- | --- | --- | --- | --- |
|  |  | FBP | IR | FBP | IR |
| Total lung volume (ml) | 4598.84±960.21 | 4582.56±995.96 | 4602.34±978.51 | 0.95 | 0.99 |
| Mean lung density (g/ml) | 0.28±0.06 | 0.28±0.06 | 0.28±0.06 | 0.83 | 0.83 |
| Lung weight (g) | 1266.25±325.01 | 1275.85±333.93 | 1281.73±332.22 | 0.90 | 0.85 |

FBP: filtered back projection

IR: iterative reconstruction
