# Supplementary material for: Quantitative assessment of Pulmonary Alveolar Proteinosis (PAP) with ultra-dose CT and correlation with Pulmonary Function Tests (PFTs)
Source: PLoS One. 2017 Mar 16;12(3):e0172958. doi: 10.1371/journal.pone.0172958 (PMC5354367; doi:10.1371/journal.pone.0172958)
Supplement: S4 Table — (DOCX) [file pone.0172958.s009.docx]

**Table 4.** Canonical loadings for input and output variates for patients with PAP

| Input set | V1 |  |  | Output set | W1 |  |  |
| --- | --- | --- | --- | --- | --- | --- | --- |
|  | LDCT | Ultra-low-dose CT | |  | LDCT | Ultra-low-dose CT | |
|  | FBP | FBP | IR |  | FBP | FBP | IR |
| FEV_1_% | 0.47 | 0.48 | 0.46 | Total lung volume | 0.36 | 0.36 | 0.36 |
| FVC% | 0.60 | 0.58 | 0.57 | Lung weight | -0.34 | -0.35 | -0.37 |
| FEV_1_/FVC | -0.10 | -0.06 | -0.07 | Mean lung density | -0.77 | -0.78 | -0.78 |
| D_LCO_% | 0.76 | 0.76 | 0.75 |  |  |  |  |
| D_LCO/_VA | 0.33 | 0.35 | -0.34 |  |  |  |  |

FBP: filtered back projection

IR: iterative reconstruction

FVC: forced vital capacity

FEV_1_: forced expiratory volume in 1 second

D_LCO_: diffusing capacity for carbon monoxide

D_LCO_/VA: diffusing capacity for carbon monoxide corrected for alveolar volume
